# Microsatellite and major histocompatibility complex variation in an endangered rattlesnake, the Eastern Massasauga (*Sistrurus catenatus*)

**DOI:** 10.1002/ece3.2159

**Published:** 2016-05-17

**Authors:** Collin P. Jaeger, Melvin R. Duvall, Bradley J. Swanson, Christopher A. Phillips, Michael J. Dreslik, Sarah J. Baker, Richard B. King

**Affiliations:** ^1^ Department of Biological SciencesNorthern Illinois UniversityDeKalbIllinois60115; ^2^ Department of BiologyCentral Michigan UniversityMt. PleasantMichigan48859; ^3^ Illinois Natural History SurveyUniversity of Illinois Urbana‐ChampaignChampaignIllinois61820

**Keywords:** Crotalinae, functional genetic variation, genetic drift, major histocompatibility complex, microsatellite, neutral genetic variation, Serpentes, Viperidae

## Abstract

Genetic diversity is fundamental to maintaining the long‐term viability of populations, yet reduced genetic variation is often associated with small, isolated populations. To examine the relationship between demography and genetic variation, variation at hypervariable loci (e.g., microsatellite DNA loci) is often measured. However, these loci are selectively neutral (or near neutral) and may not accurately reflect genomewide variation. Variation at functional trait loci, such as the major histocompatibility complex (MHC), can provide a better assessment of adaptive genetic variation in fragmented populations. We compared patterns of microsatellite and MHC variation across three Eastern Massasauga (*Sistrurus catenatus*) populations representing a gradient of demographic histories to assess the relative roles of natural selection and genetic drift. Using 454 deep amplicon sequencing, we identified 24 putatively functional MHC IIB exon 2 alleles belonging to a minimum of six loci. Analysis of synonymous and nonsynonymous substitution rates provided evidence of historical positive selection at the nucleotide level, and Tajima's D provided support for balancing selection in each population. As predicted, estimates of microsatellite allelic richness, observed, heterozygosity, and expected heterozygosity varied among populations in a pattern qualitatively consistent with demographic history and abundance. While MHC allelic richness at the population and individual levels revealed similar trends, MHC nucleotide diversity was unexpectedly high in the smallest population. Overall, these results suggest that genetic variation in the Eastern Massasauga populations in Illinois has been shaped by multiple evolutionary mechanisms. Thus, conservation efforts should consider both neutral and functional genetic variation when managing captive and wild Eastern Massasauga populations.

## Introduction

Small, isolated populations face increased risks of extinction due to complex interactions among demographic, genetic, and environmental factors (Spielman et al. 2004; Brook et al. 2008). Loss of genetic variation caused by random genetic drift can reduce survival and reproductive rates and lead to a positive feedback loop, known as an “extinction vortex” (Gilpin and Soulé 1986; Blomqvist et al. 2010). Loss of genetic diversity as a result of genetic drift may also limit a population's adaptability to future environmental changes (Spielman et al. 2004; Willi et al. 2006; Brook et al. 2008). Although genetic drift can easily overwhelm natural selection at low effective population sizes, strong balancing selection can maintain genetic variation and preserve the long‐term viability of a population. Therefore, examining the relationship between genetic variation and demographic history is a goal for conservation geneticists (Gilpin and Soulé 1986; Chapman et al. 2009; Blomqvist et al. 2010).

Advances in DNA sequencing technologies are facilitating more robust investigations into the levels and patterns of genetic variation in wild populations (Andrews and Luikart 2014). For many nonmodel species, technical limitations remain and estimates of genomic variation are often based on a few selectively neutral (or nearly neutral) markers, such as microsatellites (Vali et al. 2008). While such loci can be used to study neutral evolutionary processes (e.g., gene flow, genetic drift), they may not yield reliable estimates of overall genomic variation (Frankham 2012). In the absence of whole‐genome sequences, examination of potentially adaptive variation at functional loci can offer unique insights into the role of natural selection in wild populations.

Genes related to immunity have received considerable attention as potential adaptive trait loci (Klein 1986; Acevedo‐Whitehouse and Cunningham 2006). The major histocompatibility complex (MHC) plays an important role in the vertebrate immune system by binding and presenting antigens to T‐cells (Klein 1986). All nucleated cells express MHC class I proteins that bind endogenous peptides, whereas class II proteins are expressed only in antigen‐presenting cells and bind exogenous peptides. Variation at MHC loci is driven by balancing selection (Spurgin and Richardson 2010) and has been linked to a variety of individual‐level fitness‐related traits including reproductive success and pathogen resistance (Edwards and Hedrick 1998; Sommer 2005). At the population level, the importance of MHC variation in maintaining population viability remains poorly understood (Radwan et al. 2010). While the relevance of MHC variation in snake conservation efforts is understood (Madsen et al. 1999; Madsen and Ujvari 2006), relatively little is known regarding snake MHC evolution (Wittzell et al. 1999; Kelley et al. 2005; Ujvari and Belov 2011; Jaeger et al. 2014).

Obtaining MHC genotypes for large numbers of individuals, as required for population‐level analyses, remains logistically challenging (Babik 2010). Although conventional approaches involving cloning and Sanger sequencing may be automated, this technique is not cost‐effective at larger scales. Recently developed sequencing technologies (i.e., 454, Illumina) have been used to assess MHC variation in a variety of species, including mammals (Babik et al. 2009; Galan et al. 2010; Huchard et al. 2012; Oomen et al. 2012), birds (Zagalska‐Neubauer et al. 2010; Promerova et al. 2012; Sepil et al. 2012), turtles (Stiebens et al. 2013), and fish (Pavey et al. 2013; Lamaze et al. 2014).

The Eastern Massasauga (*Sistrurus catenatus*; ICZN 2013) is a small rattlesnake native to the Midwest and Great Lakes regions of North America. Several factors, including habitat loss and fragmentation, have contributed to declines throughout most of its range (Szymanski 1998). Today, the Eastern Massasauga is protected in both the United States (USFWS 2010) and Canada (COSEWIC 2012). In addition to wild populations, the Association of Zoos and Aquariums (AZA) maintains a captive Eastern Massasauga population as part of the Eastern Massasauga Species Survival Plan^®^. This captive population includes approximately 50 individuals representing wild populations from throughout the range of the Eastern Massasauga (Earnhardt et al. 2011; Ray et al. 2013). With a fragmented distribution and low rates of dispersal (Gibbs et al. 1997), the Eastern Massasauga represents an ideal system to examine the evolutionary processes that shape genetic variation in small, isolated populations. Previous estimates of gene flow among Eastern Massasauga populations using selectively neutral markers have demonstrated strong genetic structure at several spatial scales (Gibbs et al. 1997, 1998; Chiucchi and Gibbs 2010; DiLeo et al. 2013; Ray et al. 2013). Neutral markers have been used to estimate effective population sizes and levels of inbreeding across the range of the Eastern Massasauga (Chiucchi and Gibbs 2010). However, the relationships between genetic variation, individual fitness, and population viability remain unclear (Gibbs and Chiucchi 2012). An assessment of variation at a functional trait locus (e.g., MHC) may offer an improved understanding of the evolutionary mechanisms influencing Eastern Massasauga populations and provide valuable information for conservation efforts (Funk et al. 2012).

Our primary goal was to examine neutral and functional genetic variation in the Eastern Massasauga. First, we characterized MHC IIB exon 2 sequence variation in this species and obtained MHC genotypes for three Eastern Massasauga populations in Illinois. Next, we used these genotypes to compare levels of neutral microsatellite variation and functional MHC variation in this endangered rattlesnake. As neutral variation is influenced primarily by genetic drift, we expected that microsatellite diversity would reflect the demographic history of each population. In contrast, MHC variation may be influenced by natural selection, genetic drift, or some combination of the two. Similar patterns of MHC and microsatellite variation among these populations would indicate a major role of genetic drift on both, whereas high levels of MHC variation in each population regardless of size would provide evidence of natural selection sufficient to overcome the effects of drift.

## Methods

### Sample collection and DNA extraction

**Figure 1:**
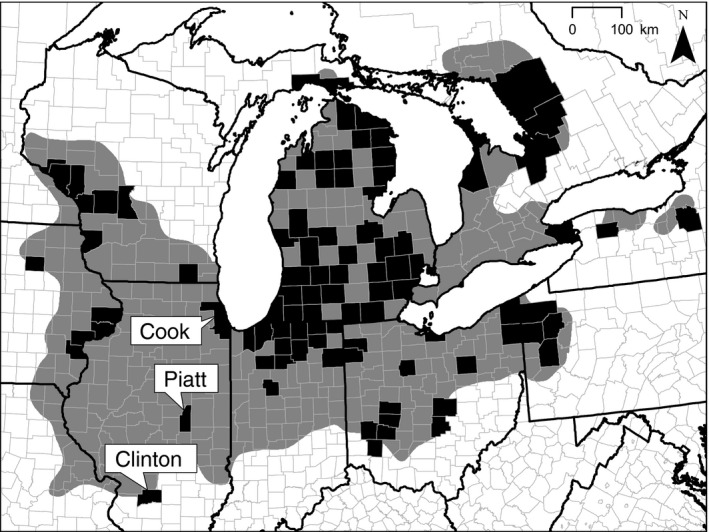
The Eastern Massasauga was historically distributed throughout the North American Great Lakes region (grey polygon). Due to a variety of factors, including habitat loss, the current distribution of this species is highly fragmented (black counties). Here, we examined genetic variation in Eastern Massasauga populations from three Illinois counties differing in terms of demographic history, from stable (Clinton) to declining (Piatt) to nearly extirpated (Cook).

We examined genetic variation in three Eastern Massasauga populations in Illinois (Fig. 1). These sites are positioned along a 400‐km transect from Clinton County to Cook County, with Piatt County centrally located ca. 170 km from Clinton County and ca. 230 km from Cook County. Although precise abundance estimates are not available, the numbers of snakes encountered during systematic and opportunistic searches demonstrate the differing demographic histories of these three populations (M. Redmer, USFWS, pers. comm., M. Dreslik, unpubl. data). In Clinton County, encounter rates based on recent (1999–2010) surveys indicate a small, but relatively stable population (0.48 snakes per search‐hour). Encounter rates are markedly lower in Piatt County (0.03 snakes per search‐hour), and no individuals were encountered in Cook County (2008–2010) indicating that this population has likely become extirpated as the samples were collected. We acknowledge that the numbers of populations and individuals likely limit our statistical power. However, these sample sizes effectively illustrate the status of the Eastern Massasauga in Illinois.

Tissue samples (i.e., blood and shed skins) were collected from Clinton County (*n *=* *89), Piatt County (*n *=* *35), and Cook County (*n *=* *10). Some of the samples were known offspring of adult female Eastern Massasaugas included in this study and were removed from population‐level analyses to minimize bias. Samples were stored in 95% ethanol at −20°C until DNA extraction. Genomic DNA was isolated using DNeasy Blood and Tissue Kits (Qiagen Inc., Valencia, CA).

### Microsatellite amplification and genotyping

We analyzed neutral genetic variation at six microsatellite loci: *Scu*‐01, *Scu*‐05, *Scu*‐07, *Scu*‐26, *Scu*‐106, *Scu*‐125 (Gibbs et al. 1998; H. L. Gibbs, pers. comm.). Amplifications were performed in 20 *μ*L volumes containing 1× GoTaq Flexi Buffer (Promega, Madison, WI), 1 U of GoTaq DNA Polymerase, 200 *μ*mol/L of each dNTP, 1.5 mmol/L of magnesium chloride (MgCl_2_), 1 *μ*mol/L of each primer, and 50–100 ng of genomic DNA. Cycling conditions were as follows: initial denaturation at 94°C for 2 min; 4 cycles of 94°C for 20 sec, a locus‐specific annealing temperature (Table S1) for 20 sec, and 72°C for 5 sec; 40 cycles of 94°C for 15 sec, the locus‐specific annealing temperature for 20 sec, and 72°C for 5 sec; and a final extension at 72°C for 2 min. Amplicons were electrophoresed on a 1% agarose gel stained with ethidium bromide, visualized under ultraviolet light, and photographed with a digital image system (Eastman Kodak, Rochester, NY). Successful reactions were analyzed using an ABI Prism 310 Genetic Analyzer (Life Technologies, Carlsbad, CA). Microsatellite genotypes were determined using GeneMapper v4 (Life Technologies).

### MHC amplification and sequencing

We used 454 deep amplicon sequencing to sequence a 166‐bp portion of MHC IIB exon 2. This gene fragment includes many of the putative antigen‐binding sites (ABS) predicted based on the human MHC IIB exon 2 protein structure (Brown et al. 1993). While the length of exon 2 is unknown in snakes, it ranges from ca. 255–270 bp in other vertebrates (Edwards and Potts 1996; Glaberman et al. 2009; Pavey et al. 2011). The combination of poor template quality and imperfectly matched degenerate PCR primers required a two‐step approach for successful amplification. First, we used degenerate primers (MHC‐UP: 5′– AAG GBC SAG TGY TAC TWY ABB AAC GG –3′; MHC‐DP: 5′– TAG TTG TGS CKG CAG WAS GTG TC –3′) to amplify MHC IIB exon 2 from genomic DNA. This primer pair targets conserved regions of the gene and has been used successfully in several reptile species (Edwards et al. 1995; Li et al. 2008), including the Eastern Massasauga (Jaeger et al. 2014). Amplifications were performed in 30 *μ*L volumes containing 1× GoTaq Flexi Buffer (Promega), 1 U of GoTaq DNA Polymerase, 200 *μ*mol/L of each dNTP, 1.5 mmol/L of magnesium chloride (MgCl_2_), 0.5 *μ*mol/L of each primer, and 50–100 ng of genomic DNA. Cycling conditions were as follows: initial denaturation at 94°C for 5 min, followed by 35 cycles of 94°C for 30 sec, 50°C for 30 sec, and 72°C for 30 sec, and a final extension at 72°C for 7 min. Amplicons were electrophoresed on a 1% agarose gel stained with ethidium bromide, visualized under ultraviolet light, and photographed with a digital image system (Eastman Kodak). Bands of the appropriate size (~215 bp) were excised and combined with 100 *μ*L of water.

In the second step, liquid from the excised bands of the first round of amplification was used as template for a second round of amplification. Here, each reaction included one of the 35 unique forward primers (Table S3). Each forward primer consisted of the Lib‐L/A adaptor, a unique 10‐bp multiplex identifier (MID), and the template‐specific sequence. Each reaction included the same reverse primer, consisting of the Lib‐L/B adaptor and the template‐specific sequence. Amplifications were performed in 30 *μ*L volumes containing 1× GoTaq Flexi Buffer (Promega), 1 U of GoTaq DNA Polymerase, 200 *μ*mol/L of each dNTP, 1.5 mmol/L of magnesium chloride (MgCl_2_), 0.5 *μ*mol/L of each primer, and 50–100 ng of genomic DNA. Cycling conditions were as follows: initial denaturation at 94°C for 5 min, followed by 30 cycles of 94°C for 30 sec, 58°C for 30 sec, and 72°C for 30 sec, and a final extension at 72°C for 7 min.

Amplicons were visualized as previously described. If a single band of the appropriate size (~300 bp) was observed, the reaction was purified using AMPure beads (Beckman Coulter Genomics, Danvers, MA). Purified amplicons were pooled at approximately equimolar concentrations, based on visual estimation on an agarose gel, and yielded five libraries, with each containing up to 21–35 unique MIDs. Each library was sequenced using Roche GS‐FLX Titanium chemistry on a ⅛ plate region by a commercial sequencing company (Macrogen Inc., Seoul, South Korea).

### MHC genotyping

We used a stepwise procedure to filter artifacts and define MHC genotypes. First, we pooled the reads (raw sequences) generated from each of the five independent 454 sequencing runs. Reads were removed if they did not contain a perfect match to the forward primer, reverse primer, and one of the supplied MID sequences. Primer and MID sequences were trimmed from each read, and the frequency of each variant (unique sequence) corresponding to each unique MID was calculated using custom scripts in R v3 (R Core Team 2013). Next, artifactual variants were distinguished from true variants within the pooled data. Each variant was queried against the NCBI nucleotide database using BLASTn (Camacho et al. 2009), and only the variants with at least one of the top ten hits corresponding to the MHC were retained. Some of these putative MHC variants may have been generated as artifacts during PCR and sequencing (Moore et al. 2006; Lenz and Becker 2008). To distinguish artifactual variants from true variants, we first assumed that an artifactual variant should be less frequent than the true variant from which it was generated. Second, each artifactual variant should be less frequent than each true variant, within a given amplicon (Babik et al. 2009; Sommer et al. 2013).

For each variant, we calculated the maximum per‐amplicon frequency (MPAF) – the highest frequency at which a particular variant was observed within any single amplicon (Zagalska‐Neubauer et al. 2010). A plot of the MPAF versus the number of reads for each variant revealed two groups: (1) a large number of variants with low MPAF and few total reads, and (2) relatively few variants with high MPAF and large numbers of total reads (Fig. S1). Thus, variants with MPAFs below 0.10 (10%) were considered to be artifacts and were removed, while variants with MPAFs above this value were considered to be putative MHC alleles in subsequent analyses. In the final step, artifactual variants were separated from true variants within individual amplicons. Here, a variant was considered true within an amplicon if its frequency was ≥0.10 (10%) of the most frequent variant within that amplicon (Stiebens et al. 2013). This approach is likely conservative and may underestimate the number of MHC alleles possessed by a given individual. However, as it was applied consistently to individuals from each population, we do not expect this approach to bias our overall interpretation. Additional details regarding the verification of MHC genotypes are provided in the supplemental materials (see Appendix S1 for more details).

### MHC sequence analysis

The putative Eastern Massasauga MHC nucleotide sequences were aligned using MUSCLE v3 (Edgar 2004). Pairwise uncorrected amino acid *p*‐distances and Tamura–Nei genetic distances were calculated using MEGA v5 (Tamura et al. 2011). For each individual, we estimated MHC variation in two ways. First, we determined the number of MHC alleles per individual. Second, we calculated nucleotide diversity based on individual MHC genotypes using the “nuc.div” function as implemented in the “pegas” package (Paradis 2010) in R v3 (R Core Team 2013).

To test for positive selection, we estimated the relative rate of nonsynonymous and synonymous substitutions (*d*N/*d*S). Although this approach was originally designed to test for positive selection based on fixed differences among species (Kryazhimskiy and Plotkin 2008), it is commonly implemented to measure MHC variation within populations (Hughes and Nei 1989; Bernatchez and Landry 2003; Garrigan and Hedrick 2003). First, we calculated *d*N/*d*S using MEGA v5. We specified the putative antigen‐binding sites a priori based on the human MHC IIB exon 2 structure (Brown et al. 1993) and ran three separate analyses: (1) the putative antigen‐binding sites (ABS), (2) non‐antigen‐binding sites (non‐ABS), and (3) the total sequence. For each analysis, we used the modified Nei–Gojobori method with Jukes–Cantor correction (Nei and Gojobori 1986). Standard errors were generated based on 1000 replicates, and rates were compared using a *Z*‐test. Second, we compared evolutionary models of *d*N/*d*S in a maximum‐likelihood framework using CODEML as implemented in PAML v4 (Yang 2007). We used likelihood ratio tests to compare two sets of nested models. The first test compared models M1a (neutral) and M2a (selection). Models M1a and M2a both allow for the strength of selection to vary across sites. However, model M1a does not allow for positive selection, while model M2a does. The second test compared models M7 (beta) and M8 (beta and omega). Models M7 and M8 both use the beta distribution to allow the strength of selection to vary across sites. However, model M7 does not allow for positive selection, while model M8 does. Results of the likelihood ratio tests were compared to a chi‐square distribution with *α *= 0.05 and degrees of freedom equal to the difference in the number of parameters between the nested models. Positively selected sites were identified using Bayes Empirical Bayes analysis (Yang et al. 2005).

### Measures of neutral and functional genetic diversity

To estimate population‐level microsatellite variation, we calculated rarefied allelic richness and expected heterozygosity for each population using MSA v4 (Dieringer and Schlotterer 2003). Expected heterozygosity is a robust estimator of microsatellite variation within a population when sample size is small (Gorman and Renzi 1979). Due to differences in sample sizes, statistical comparisons among the three populations were made using randomization tests implemented in R. Based on the MHC sequence data, we calculated nucleotide diversity (*π*) and Tajima's D for each population using the “pegas” package in R (Paradis 2010). We also calculated MHC allelic richness for each population, after rarefying for unequal sample sizes.

At the individual level, we calculated multilocus heterozygosity as the number of heterozygous loci divided by the number of genotyped loci per individual. We defined individual‐level MHC variation as the number and nucleotide diversity of alleles possessed by an individual. We used Spearman's rank correlations to test for relationships between (1) microsatellite multilocus heterozygosity and the number of MHC alleles and (2) microsatellite multilocus heterozygosity and MHC nucleotide diversity.

## Results

### MHC characterization

We obtained 729,085 reads from five 454 sequencing runs. Of these, 374,399 usable MHC reads were identified, consisting of 751 unique sequence variants. The remaining 354,686 reads were discarded because they (1) did not contain both primer sequences and one of the MID sequences or (2) were not identified as MHC based on BLAST analysis. The number of usable MHC reads per sample was high, but variable (mean ± SD = 2.214 ± 1.603, range = 105–7192) when excluding four samples with poor sequencing depth. Of the 751 unique MHC variants, only 25 had maximum per‐amplicon frequencies ≥0.10 (10%) and were considered putative alleles (Fig. S1, Appendix S1). While these 25 putative alleles represented less than 5% of the unique variants (25 of 751 variants), they accounted for approximately 75% of the usable MHC reads (280,741 of 374,399 reads).

Of the 25 putative alleles, five alleles (*Sica*‐DAB*01–*04, *07) were previously identified (Jaeger et al. 2014). The 20 newly identified putative alleles were named *Sica*‐DAB*10–29, according to the nomenclatural standards (Klein et al. 1990). Each putative allele was 166 bp in length, except *Sica*‐DAB*10, which was 153 bp due to a 13‐bp deletion (positions 111–123) leading to a frameshift. This sequence occurred in Clinton County, where it was present in >70% of individuals. However, *Sica*‐DAB*10 likely represents a pseudogene and was excluded from subsequent phylogenetic comparisons and tests of selection. The variation in the number of putative MHC alleles within individuals was not an artifact of read depth because it was not associated with the number of reads within individuals (*n *=* *169, *r *= −0.063, *P *=* *0.416; Fig. S2, Appendix S1).

**Figure 2:**
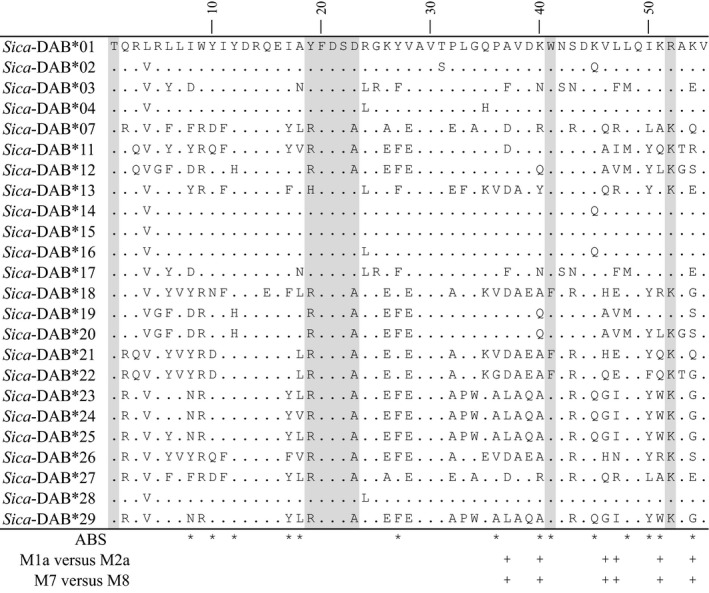
Amino acid alignment of MHC IIB exon 2 alleles showing evidence of positive selection (indicated by + signs). Shaded sites correspond to conserved residues typically found in MHC IIB molecules. Asterisks indicate putative antigen‐binding sites (ABS) identified based on homology to the human sequence (Brown et al. 1993).

Pairwise comparisons of the 24 putatively functional alleles revealed high levels of variation. Ninety of the 166 (54.2%) nucleotide positions were variable with pairwise Tamura–Nei genetic distances ranging from 0.6 to 57.6% (mean ± SD = 31.9 ± 15.2%). The 24 unique nucleotide sequences translated into 22 unique amino acid sequences (Fig. 2). Forty‐three of the 55 (78.2%) amino acid positions were variable with pairwise uncorrected *p*‐distances ranging from 1.8 to 58.2% (mean ± SD = 36.9 ± 14.9%). We found evidence of positive selection acting on the entire sequence (*d*N/*d*S = 1.90, *Z *=* *2.89, *P *=* *0.002; Table 1). The strength of positive selection was higher in the putative antigen‐binding sites (ABS; *d*N/*d*S = 2.76, *Z *=* *2.99, *P *=* *0.002) when compared to the rest of the sequence (non‐ABS; *d*N/*d*S = 1.57, *Z *=* *1.52, *P *=* *0.066). Maximum‐likelihood analyses also revealed evidence of positive selection (Fig. 2). Model M2a was favored over model M1a (*LR* = 31.81, df = 2, *P *<* *0.0001), and model M8 was favored over model M7 (*LR* = 36.24, df = 2, *P *<* *0.0001). Bayes Empirical Bayes analyses identified six positively selected sites when comparing both sets of nested models: sites 37, 40, 46, 47, 51, 54 (Fig. 2). Three of the six positively selected sites (40, 51, 54) correspond to antigen‐binding sites of the human MHC. However, we did not find evidence for positive selection at 11 other sites corresponding to antigen‐binding sites in the human MHC.

**Table 1:** Rates of synonymous and nonsynonymous substitutions indicate that Eastern Massasauga MHC IIB exon 2 sequences are influenced by strong positive selection, with much of this selection focused on the putative antigen‐binding sites (ABS)

	*d*_N_	*d*_S_	*d*_N_/*d*_S_	*Z*‐score	*P*‐value
Total	0.298 ± 0.046	0.157 ± 0.034	1.90	2.89	0.002
ABS	0.650 ± 0.154	0.236 ± 0.095	2.76	2.99	0.002
non‐ABS	0.213 ± 0.040	0.136 ± 0.038	1.57	1.52	0.066

*d*_N_, nonsynonymous substitution rate; *d*_S_, synonymous substitution rate.

### Measures of neutral and functional genetic variation

We obtained genotypes for 134 Eastern Massasaugas from three Illinois counties at six microsatellite loci (Table 2). For all microsatellite analyses, known offspring were excluded, yielding the following sample sizes: Clinton County (*n *=* *89), Piatt County (*n *=* *17), Cook County (*n *=* *6). Piatt County showed intermediate levels of rarefied allelic richness and expected heterozygosity compared with Cook and Clinton counties. However, randomization tests revealed significant differences (*P *<* *0.05) only between Cook and Clinton counties, with Clinton County exceeding Cook County in both measures.

**Table 2:** Summary of neutral and functional genetic variation for Eastern Massasaugas in Illinois based on six microsatellite loci and partial coding sequence of the major histocompatibility complex (MHC) IIB exon 2. Values in parentheses represent standard errors (SE) unless otherwise noted

	Clinton county	Piatt county	Cook county
Microsatellites			
Sample size, *n*	89	17	6
Allelic richness	7.00 (0.58)	5.00 (0.89)	2.83 (0.40)
Rarefied allelic richness	3.83 (0.13)	3.43 (0.52)	2.83 (0.40)
Observed heterozygosity	0.51 (0.03)	0.50 (0.05)	0.44 (0.13)
Expected heterozygosity	0.66 (0.03)	0.66 (0.05)	0.44 (0.09)
MHC IIB exon 2			
Sample size, *n*	79	17	6
Tajima's D (*P‐*value)	3.620 (0.003)	2.090 (0.037)	2.330 (0.020)
Allelic richness	18	11	8
Rarefied allelic richness	11.64 (1.82)	9.15 (1.13)	8
Nucleotide diversity, *π*	0.188 (0.091)	0.172 (0.084)	0.216 (0.110)
Mean alleles per individual	4.71 (0.22)	4.12 (0.19)	3.17 (0.31)

We found 18 putative MHC alleles in Clinton County (including seven private alleles), 11 alleles in Piatt County (including one private allele), and eight alleles in Cook County (no private alleles; Table 2). One allele (*Sica*‐DAB*03) was nearly ubiquitous, occurring in all but five individuals. We were not able to determine MHC genotypes for ten individuals from Clinton County, so MHC analyses were based on the following sample sizes: Clinton County (*n *=* *79), Piatt County (*n *=* *17), Cook County (*n *=* *6). In each population, Tajima's D was significantly greater than one: Clinton County (D = 3.62, *P *<* *0.01), Piatt County (D = 2.09, *P *<* *0.05), Cook County (D = 2.33, *P *<* *0.05). Eastern Massasaugas possessed between one and 12 MHC alleles, indicating the presence of at least six loci. The mean number of MHC alleles per individual was lowest in Cook County, intermediate in Piatt County, and highest in Clinton County. At the population level, MHC nucleotide diversity was higher in Cook County (*π * =  0.216) than in Clinton County (*π * =  0.188) or Piatt County (*π * =  0.172).

We found no evidence of any relationship between microsatellite and MHC variation within individuals. Multilocus microsatellite heterozygosity was not significantly correlated with MHC allelic richness (*n *=* *102, *r *= −0.048, *P *=* *0.633) or MHC nucleotide diversity (*n *=* *102, *r *= −0.077, *P *=* *0.445).

## Discussion

### Characterization of Eastern Massasauga MHC IIB exon 2

As predicted, the 24 putatively functional Eastern Massasauga MHC alleles were highly variable, at both the nucleotide and amino acid levels. Consistent with previous work (Jaeger et al. 2014), we found an excess of nonsynonymous substitutions in the Eastern Massasauga MHC, indicating historical positive selection. Although this signature of selection was higher on the putative antigen‐binding sites than the non‐antigen‐binding sites predicted based on the human MHC protein structure (Brown et al. 1993), only three of the 14 amino acid residues predicted to bind antigens were identified as positively selected sites based on the maximum‐likelihood analysis of evolutionary models. Also, three positively selected sites were adjacent to putative antigen‐binding sites. This discrepancy may indicate that (1) not all of the antigen‐binding sites are positively selected in the Eastern Massasauga or (2) the Eastern Massasauga MHC IIB protein is structurally different from its human homolog. Similar discrepancies occur with other species, including the Eurasian Coot (*Fulica atra*; Alcaide et al. 2014), the Japanese Black Bear (*Ursus thibetanus*; Yasukochi et al. 2012), and the Grey Partridge (*Perdix perdix*; Promerova et al. 2013).

The maintenance of multiple MHC alleles is likely the result of balancing selection mediated by host–pathogen interactions (Edwards and Hedrick 1998; Spurgin and Richardson 2010). Three mechanisms have been proposed to explain the high polymorphism of MHC loci: (1) heterozygote advantage, (2) negative frequency‐dependent selection, and (3) fluctuating selection. These mechanisms are not mutually exclusive and may produce similar patterns of MHC variation, making it difficult to distinguish among them in natural populations (van Oosterhout 2009). The amount of MHC diversity in these Eastern Massasauga populations is similar to estimates reported for the Tuatara (*Sphenodon punctatus*; Miller et al. 2010) and the Ornate Dragon Lizard (*Ctenophorus ornatus*; Radwan et al. 2014).

We found a considerable variation in the number of MHC alleles identified within individual Eastern Massasaugas. Individuals possessed 1–12 alleles, suggesting a minimum of 6 loci. While this variation may be influenced by differences in the quality of DNA templates used during library preparation (Taberlet et al. 1996; Lighten et al. 2014a), we found no evidence of systematic biases that would potentially skew comparisons among populations. This result suggests that either (1) copy number varies among individuals or (2) some alleles are shared across loci (Eimes et al. 2011; Radwan et al. 2012). Similar copy number variation has been documented in mammals (Galan et al. 2010; Siddle et al. 2010; Oomen et al. 2012), birds (Zagalska‐Neubauer et al. 2010; Eimes et al. 2011; Promerova et al. 2012; Radwan et al. 2012), turtles (Stiebens et al. 2013), and fish (Lighten et al. 2014b). This pattern has also been linked to susceptibility to a contagious cancer in the Tasmanian Devil (*Sarcophilus harrisii*; Siddle et al. 2010) and body condition in the Loggerhead Sea Turtle (*Caretta caretta*; Stiebens et al. 2013). Additional work is needed to clarify what, if any, relationship exists between MHC copy number and phenotypic fitness in the Eastern Massasauga.

### Comparison of neutral and functional variation

The amount of neutral genetic variation within a population is influenced by random evolutionary processes (i.e., genetic drift, gene flow, mutation) and is positively associated with effective population size (Frankham 1996). In addition to these random evolutionary processes, the amount of adaptive variation within a population can be influenced by natural selection (Piertney and Oliver 2006; Sutton et al. 2011). In some cases, MHC variation has been maintained despite severe population bottlenecks and reductions in microsatellite variation. Natural selection can prevail over genetic drift, even in small populations (Aguilar et al. 2004; Babik et al. 2008; Biedrzycka and Radwan 2008; Oliver and Piertney 2012; Vásquez‐Carrillo et al. 2014), but this is not always the case in species with fragmented distributions, such as the Eurasian Beaver (*Castor fiber*; Babik et al. 2005), Black Grouse (*Tetrao tetrix*; Strand et al. 2012), and Tuatara (*Sphenodon punctatus*; Miller et al. 2010).

These Eastern Massasauga populations in Illinois represent a gradient of demographic histories: from relatively stable in Clinton County to declining in Piatt County to likely extirpated in Cook County (M. Dreslik, unpubl. data). We found a general positive relationship between demographic history and microsatellite variation. Despite low statistical power, we found significant differences between Clinton and Cook counties in terms of microsatellite‐rarefied allelic richness and expected heterozygosity. This is likely due to the effects of inbreeding and genetic drift in this small population. Overall, these results are comparable to those reported previously for this species (Chiucchi and Gibbs 2010). Although our sample sizes were low in Piatt and Cook Counties, our samples represent nearly all known individuals from these populations in recent years (excluding known offspring). More robust estimates of neutral genetic variation would require additional data from additional individuals and loci.

In each population, estimates of Tajima's D based on the MHC sequences were significantly greater than zero indicating balancing selection or population contraction (Tajima 1989). This finding is consistent with previous analyses of synonymous and nonsynonymous mutation rates in the Eastern Massasauga MHC (Jaeger et al. 2014). Although we found a great deal of variation, the number of MHC alleles per individual showed an increasing trend from Cook to Piatt and Clinton counties, consistent with our microsatellite results. This suggests that individual MHC genotypes may be more strongly influenced by genetic drift than selection. At the population level, MHC nucleotide diversity was unexpectedly high in Cook County, indicating that this smaller population contains fewer, yet more divergent, MHC alleles.

We found no significant relationship between microsatellite and MHC variation among individual Eastern Massasaugas, suggesting that neither set of markers may accurately represent overall genomic variation in this species (Vali et al. 2008). Although we did not quantify fitness per se, previous research found no correlation between microsatellite variation and body condition in the Eastern Massasauga (Gibbs and Chiucchi 2012). In contrast, greater MHC variation in the Water Python (*Liasis fuscus*) was associated with reduced parasitism and increased longevity (Madsen and Ujvari 2006). Similar relationships have been documented for a variety of other species (Klein and O'huigin 1994; Sommer 2005). Future work should investigate associations between MHC variation and proxies of individual fitness, including reproductive success, pathogen resistance (Madsen and Ujvari 2006), and juvenile growth rate (Madsen and Shine 2000) in the Eastern Massasauga.

In summary, we find evidence consistent with the effects of both genetic drift and natural selection in influencing genetic variation in the Eastern Massasauga in Illinois. On historical timescales, synonymous and nonsynonymous nucleotide substitution rates reveal the effects of strong positive selection. However, on a more recent timescale, the diversity of microsatellite and MHC alleles among these populations suggests a role for genetic drift (Mainguy et al. 2007; Miller et al. 2008; Babik et al. 2012; Strand et al. 2012). Our results should be interpreted cautiously, however, due to our limited sample sizes (both in terms of the number of populations and the availability of samples).

We found a relatively high level of MHC nucleotide diversity in the smallest population (Cook County). Although this population is believed to be extirpated at this time, future work should examine other small and declining populations to determine whether this result could be an indication of increased selective pressure as the population declines (Spurgin and Richardson 2010). Future studies should also assess (1) neutral and functional genetic variation in additional Eastern Massasauga populations and (2) the relationship, if any, between genetic diversity, phenotypic fitness, and population viability. Collectively, these results provide an estimate of functional genetic variation and help establish a genetic baseline for this endangered species. Such a baseline will inform and guide ongoing Eastern Massasauga conservation efforts, including the captive breeding program (Earnhardt et al. 2011). In light of emerging fungal pathogens affecting snake populations, future work should examine the relationship between genetic variation and pathogen resistance (Bertelsen et al. 2005; Allender et al. 2013).

## Conflict of Interest

None declared.

## Data Accessibility

Dryad: doi:10.5061/dryad.s6f76.

## Supporting information


**Appendix S1.** Verification of MHC Genotypes.
**Table S1.** Characteristics of microsatellite DNA loci used to measure neutral genetic variation in the Eastern Massasauga (Gibbs et al. 1998; H. L. Gibbs, pers. comm.).
**Table S2.** Microsatellite alleles and allele frequencies for Eastern Massasaugas in (A) Clinton County, (B) Piatt County, and (C) Cook County, Illinois. Shading indicates private alleles.
**Table S3.** Primer sequences used for PCR amplification and 454 sequencing of MHC IIB exon 2 in the Eastern Massasauga. Adaptor sequences are in blue, multiplex identifiers are in red, and template‐specific sequences are in black.
**Table S4.** Summary of replicated MHC IIB exon 2 genotypes (*n *=* *20) determined via 454 sequencing in the Eastern Massasauga.
**Table S5.** Comparison of Eastern Massasauga MHC IIB exon 2 genotypes in three individuals based on cloning and Sanger sequencing and 454 sequencing. Alleles identified by both methods for each individual are highlighted.
**Table S6.** MHC IIB exon 2 genotypes of three Eastern Massasauga mothers and their offspring. The prefix *‘Sica*‐DAB*’ is omitted from allele names for brevity. The presence of a given maternal allele in an offspring genotype is indicated with a plus sign (+). Putative paternal alleles (alleles not found in mothers) are also listed for each offspring.
**Figure S1.** Eastern Massasauga MHC IIB exon 2 variants with maximum per‐amplicon frequencies above 0.10 (10%; red dotted line) were considered putatively true alleles, while those below this threshold were treated as artefacts and excluded from subsequent analyses.
**Figure S2.** The number of MHC IIB exon 2 alleles was not significantly correlated with sequencing depth (number of reads) per individual (*n *=* *169, *r *= −0.063, *P *=* *0.416).
