# Supplementary material for: Microsatellite and major histocompatibility complex variation in an endangered rattlesnake, the Eastern Massasauga (Sistrurus catenatus)
Source: Ecol Evol. 2016 May 17;6(12):3991–4003. doi: 10.1002/ece3.2159 (PMC4874855; doi:10.1002/ece3.2159)
Supplement: Supplementary file 1 — Appendix S1. Verification of MHC Genotypes. Table S1. Characteristics of microsatellite DNA loci used to measure neutral genetic variation in the Eastern Massasauga (Gibbs et al. 1998; H. L. Gibbs, pers. comm.). Table S2. Microsatellite alleles and allele frequencies for Eastern Massasaugas in (A) Clinton County, (B) Piatt County, and (C) Cook County, Illinois. Shading indicates private alleles. Table S3. Primer sequences used for PCR amplification and 454 sequencing of MHC IIB exon 2 in the Eastern Massasauga. Adaptor sequences are in blue, multiplex identifiers are in red, and template‐specific sequences are in black. Table S4. Summary of replicated MHC IIB exon 2 genotypes (n = 20) determined via 454 sequencing in the Eastern Massasauga. Table S5. Comparison of Eastern Massasauga MHC IIB exon 2 genotypes in three individuals based on cloning and Sanger sequencing and 454 sequencing. Alleles identified by both methods for each individual are highlighted. Table S6. MHC IIB exon 2 genotypes of three Eastern Massasauga mothers and their offspring. The prefix ‘Sica‐DAB*’ is omitted from allele names for brevity. The presence of a given maternal allele in an offspring genotype is indicated with a plus sign (+). Putative paternal alleles (alleles not found in mothers) are also listed for each offspring. Figure S1. Eastern Massasauga MHC IIB exon 2 variants with maximum per‐amplicon frequencies above 0.10 (10%; red dotted line) were considered putatively true alleles, while those below this threshold were treated as artefacts and excluded from subsequent analyses. Figure S2. The number of MHC IIB exon 2 alleles was not significantly correlated with sequencing depth (number of reads) per individual (n = 169, r = −0.063, P = 0.416). [file ECE3-6-3991-s001.docx]

**Table S1.** Characteristics of microsatellite DNA loci used to measure neutral genetic variation in the Eastern Massasauga (Gibbs *et al.* 1998; H.L. Gibbs, pers. comm.).

| **Locus** | **Primer sequence (5'–3')** | ***T_A_*** | **Size range (bp)** | ***AR*** | ***H_O_*** |
| --- | --- | --- | --- | --- | --- |
| *Scu*-01 | F: GTCAACACTTGTGTTCTGC  R: [5HEX]CTGTATTAAAGTTGTTTTGTTCA | 51 | 135–168 | 11 | 0.49 |
| *Scu*-05 | F: GACATTGCTGAACAGACTAT  R: [5HEX]TTGTGTAGCATAGTGAAACA | 54 | 168–211 | 9 | 0.44 |
| *Scu*-07 | F: CTTTGTGCTATTTTTCCACC  R: [5TET]GCCAAAAAAGTAAAATATGAGC | 59 | 152–184 | 9 | 0.46 |
| *Scu*-26 | F: GAAATTGGTGGAAGAGACCTG  R: [5TET]GTCCAGGATATGAGGGATCTG | 59 | 167–181 | 7 | 0.63 |
| *Scu*-106 | F: [6FAM]AAGAGCCAATACTGGGGTCC  R: CTGGATGGCGAATCCAAAC | 58 | 115–127 | 6 | 0.54 |
| *Scu*-125 | F: [6FAM]GTCAACCCATCACGACTC  R: TTTCTCTGCTATGCAATATCC | 56 | 162–207 | 7 | 0.48 |

*T_A_* = annealing temperature (ºC), bp = base pairs, *AR* = allelic richness, *H_O_* = observed heterozygosity

**Table S2.** Microsatellite alleles and allele frequencies for Eastern Massasaugas in (A) Clinton County, (B) Piatt County, and (C) Cook County, Illinois. Shading indicates private alleles.

| **(A) Clinton County** | | | | | | | | | | | |
| --- | --- | --- | --- | --- | --- | --- | --- | --- | --- | --- | --- |
| ***Scu*-01** | | ***Scu*-05** | | ***Scu*-07** | | ***Scu*-26** | | ***Scu*-106** | | ***Scu*-125** | |
| **(*n* = 87)** | | **(*n* = 87)** | | **(*n* = 86)** | | **(*n* = 88)** | | **(*n* = 89)** | | **(*n* = 85)** | |
| 135 | 0.155 | 168 | 0.046 | 166 | 0.058 | 167 | 0.017 | 115 | 0.011 | 162 | 0.006 |
| 141 | 0.006 | 185 | 0.006 | 168 | 0.314 | 173 | 0.307 | 121 | 0.208 | 170 | 0.006 |
| 145 | 0.080 | 188 | 0.534 | 170 | 0.047 | 175 | 0.244 | 123 | 0.129 | 189 | 0.594 |
| 147 | 0.420 | 193 | 0.006 | 172 | 0.465 | 177 | 0.182 | 125 | 0.562 | 191 | 0.076 |
| 150 | 0.276 | 195 | 0.075 | 174 | 0.099 | 179 | 0.011 | 127 | 0.090 | 195 | 0.059 |
| 154 | 0.052 | 197 | 0.282 | 176 | 0.006 | 181 | 0.239 |  |  | 203 | 0.247 |
| 163 | 0.011 | 216 | 0.034 | 180 | 0.006 |  |  |  |  | 207 | 0.012 |
|  |  | 219 | 0.011 | 184 | 0.006 |  |  |  |  |  |  |
|  |  | 221 | 0.006 |  |  |  |  |  |  |  |  |
|  |  |  |  |  |  |  |  |  |  |  |  |
| **(B) Piatt County** | | | | | | | | | | | |
| ***Scu*-01** | | ***Scu*-05** | | ***Scu*-07** | | ***Scu*-26** | | ***Scu*-106** | | ***Scu*-125** | |
| **(*n* = 17)** | | **(*n* = 17)** | | **(*n* = 17)** | | **(*n* = 17)** | | **(*n* = 17)** | | **(*n* = 17)** | |
| 135 | 0.265 | 188 | 0.34 | 166 | 0.294 | 171 | 0.265 | 119 | 0.294 | 189 | 0.382 |
| 143 | 0.206 | 216 | 0.66 | 168 | 0.382 | 173 | 0.353 | 123 | 0.118 | 195 | 0.471 |
| 145 | 0.265 |  |  | 172 | 0.324 | 175 | 0.059 | 125 | 0.588 | 203 | 0.147 |
| 147 | 0.029 |  |  |  |  | 177 | 0.235 |  |  |  |  |
| 150 | 0.059 |  |  |  |  | 181 | 0.088 |  |  |  |  |
| 152 | 0.088 |  |  |  |  |  |  |  |  |  |  |
| 163 | 0.029 |  |  |  |  |  |  |  |  |  |  |
| 168 | 0.059 |  |  |  |  |  |  |  |  |  |  |
|  |  |  |  |  |  |  |  |  |  |  |  |
| **(C) Cook County** | | | | | | | | | | | |
| ***Scu*-01** | | ***Scu*-05** | | ***Scu*-07** | | ***Scu*-26** | | ***Scu*-106** | | ***Scu*-125** | |
| **(*n* = 6)** | | **(*n* = 6)** | | **(*n* = 6)** | | **(*n* = 6)** | | **(*n* = 6)** | | **(*n* = 6)** | |
| 135 | 0.083 | 188 | 0.08 | 152 | 0.083 | 171 | 0.083 | 121 | 0.417 | 189 | 0.083 |
| 154 | 0.333 | 195 | 0.92 | 172 | 0.333 | 173 | 0.083 | 125 | 0.583 | 195 | 0.917 |
| 156 | 0.583 |  |  | 174 | 0.417 | 177 | 0.083 |  |  |  |  |
|  |  |  |  | 176 | 0.167 | 181 | 0.750 |  |  |  |  |
|  |  |  |  |  |  |  |  |  |  |  |  |

**Table S3.** Primer sequences used for PCR amplification and 454 sequencing of MHC IIB exon 2 in the Eastern Massasauga. Adaptor sequences are in blue, multiplex identifiers are in red, and template-specific sequences are in black.

| **Name** | **Sequence (5'–3')** |
| --- | --- |
| ***Forward primers*** |  |
| MHC-UP | AAGGBCSAGTGYTACTWYABBAACGG |
| 454MHC-UP-MID01 | CCATCTCATCCCTGCGTGTCTCCGACTCAGACGAGTGCGTAAGGBCSAGTGYTACTWYABBAACGG |
| 454MHC-UP-MID02 | CCATCTCATCCCTGCGTGTCTCCGACTCAGACGCTCGACAAAGGBCSAGTGYTACTWYABBAACGG |
| 454MHC-UP-MID03 | CCATCTCATCCCTGCGTGTCTCCGACTCAGAGACGCACTCAAGGBCSAGTGYTACTWYABBAACGG |
| 454MHC-UP-MID04 | CCATCTCATCCCTGCGTGTCTCCGACTCAGAGCACTGTAGAAGGBCSAGTGYTACTWYABBAACGG |
| 454MHC-UP-MID05 | CCATCTCATCCCTGCGTGTCTCCGACTCAGATCAGACACGAAGGBCSAGTGYTACTWYABBAACGG |
| 454MHC-UP-MID06 | CCATCTCATCCCTGCGTGTCTCCGACTCAGATATCGCGAGAAGGBCSAGTGYTACTWYABBAACGG |
| 454MHC-UP-MID07 | CCATCTCATCCCTGCGTGTCTCCGACTCAGCGTGTCTCTAAAGGBCSAGTGYTACTWYABBAACGG |
| 454MHC-UP-MID08 | CCATCTCATCCCTGCGTGTCTCCGACTCAGCTCGCGTGTCAAGGBCSAGTGYTACTWYABBAACGG |
| 454MHC-UP-MID09 | CCATCTCATCCCTGCGTGTCTCCGACTCAGTAGTATCAGCAAGGBCSAGTGYTACTWYABBAACGG |
| 454MHC-UP-MID10 | CCATCTCATCCCTGCGTGTCTCCGACTCAGTCTCTATGCGAAGGBCSAGTGYTACTWYABBAACGG |
| 454MHC-UP-MID11 | CCATCTCATCCCTGCGTGTCTCCGACTCAGTGATACGTCTAAGGBCSAGTGYTACTWYABBAACGG |
| 454MHC-UP-MID12 | CCATCTCATCCCTGCGTGTCTCCGACTCAGTACTGAGCTAAAGGBCSAGTGYTACTWYABBAACGG |
| 454MHC-UP-MID13 | CCATCTCATCCCTGCGTGTCTCCGACTCAGCATAGTAGTGAAGGBCSAGTGYTACTWYABBAACGG |
| 454MHC-UP-MID14 | CCATCTCATCCCTGCGTGTCTCCGACTCAGCGAGAGATACAAGGBCSAGTGYTACTWYABBAACGG |
| 454MHC-UP-MID15 | CCATCTCATCCCTGCGTGTCTCCGACTCAGATACGACGTAAAGGBCSAGTGYTACTWYABBAACGG |
| 454MHC-UP-MID16 | CCATCTCATCCCTGCGTGTCTCCGACTCAGTCACGTACTAAAGGBCSAGTGYTACTWYABBAACGG |
| 454MHC-UP-MID17 | CCATCTCATCCCTGCGTGTCTCCGACTCAGCGTCTAGTACAAGGBCSAGTGYTACTWYABBAACGG |
| 454MHC-UP-MID18 | CCATCTCATCCCTGCGTGTCTCCGACTCAGTCTACGTAGCAAGGBCSAGTGYTACTWYABBAACGG |
| 454MHC-UP-MID19 | CCATCTCATCCCTGCGTGTCTCCGACTCAGTGTACTACTCAAGGBCSAGTGYTACTWYABBAACGG |
| 454MHC-UP-MID21 | CCATCTCATCCCTGCGTGTCTCCGACTCAGCGTAGACTAGAAGGBCSAGTGYTACTWYABBAACGG |
| 454MHC-UP-MID22 | CCATCTCATCCCTGCGTGTCTCCGACTCAGTACGAGTATGAAGGBCSAGTGYTACTWYABBAACGG |
| 454MHC-UP-MID23 | CCATCTCATCCCTGCGTGTCTCCGACTCAGTACTCTCGTGAAGGBCSAGTGYTACTWYABBAACGG |
| 454MHC-UP-MID24 | CCATCTCATCCCTGCGTGTCTCCGACTCAGTAGAGACGAGAAGGBCSAGTGYTACTWYABBAACGG |
| 454MHC-UP-MID25 | CCATCTCATCCCTGCGTGTCTCCGACTCAGTCGTCGCTCGAAGGBCSAGTGYTACTWYABBAACGG |
| 454MHC-UP-MID26 | CCATCTCATCCCTGCGTGTCTCCGACTCAGACATACGCGTAAGGBCSAGTGYTACTWYABBAACGG |
| 454MHC-UP-MID27 | CCATCTCATCCCTGCGTGTCTCCGACTCAGACGCGAGTATAAGGBCSAGTGYTACTWYABBAACGG |
| 454MHC-UP-MID28 | CCATCTCATCCCTGCGTGTCTCCGACTCAGACTACTATGTAAGGBCSAGTGYTACTWYABBAACGG |
| 454MHC-UP-MID29 | CCATCTCATCCCTGCGTGTCTCCGACTCAGACTGTACAGTAAGGBCSAGTGYTACTWYABBAACGG |
| 454MHC-UP-MID30 | CCATCTCATCCCTGCGTGTCTCCGACTCAGAGACTATACTAAGGBCSAGTGYTACTWYABBAACGG |
| 454MHC-UP-MID31 | CCATCTCATCCCTGCGTGTCTCCGACTCAGAGCGTCGTCTAAGGBCSAGTGYTACTWYABBAACGG |
| 454MHC-UP-MID32 | CCATCTCATCCCTGCGTGTCTCCGACTCAGAGTACGCTATAAGGBCSAGTGYTACTWYABBAACGG |
| 454MHC-UP-MID33 | CCATCTCATCCCTGCGTGTCTCCGACTCAGATAGAGTACTAAGGBCSAGTGYTACTWYABBAACGG |
| 454MHC-UP-MID34 | CCATCTCATCCCTGCGTGTCTCCGACTCAGCACGCTACGTAAGGBCSAGTGYTACTWYABBAACGG |
| 454MHC-UP-MID35 | CCATCTCATCCCTGCGTGTCTCCGACTCAGCAGTAGACGTAAGGBCSAGTGYTACTWYABBAACGG |
| ***Reverse primers*** |  |
| MHC-DP | TAGTTGTGSCKGCAGWASGTGTC |
| 454MHC-DP | CCTATCCCCTGTGTGCCTTGGCAGTCTCAGTAGTTGTGSCKGCAGWASGTGTC |

**Appendix 1: Verification of MHC Genotypes**

We evaluated the validity of the MHC genotypes in three ways. First, for a sample of individuals (*n* = 20), we compared two independent MHC genotypes via 454 sequencing. Second, we compared MHC genotypes derived from 454 sequencing with those previously obtained via cloning and Sanger sequencing for three individuals (Jaeger *et al.* 2014). Finally, we compared MHC genotypes among known relatives (i.e., dams and offspring) to examine the degree to which the transmission of MHC alleles was consistent with a Mendelian pattern of inheritance.

First, we compared replicated genotypes obtained independently for 20 individuals (Table S4). Of these, genotypes for eleven individuals were identical across replicates, including one individual possessing seven alleles. Genotypes for three additional individuals were nearly identical across replicates, differing by a single allele. In each case, a low-frequency allele present in both replicates did not meet the individual threshold in one of the two replicates. Genotypes for four additional individuals were similar, with 2–4 shared alleles and 2–3 unshared alleles across replicates. Genotypes for the remaining two individuals were quite different, with no alleles shared across replicates. Next, we compared MHC genotypes derived from 454 sequencing with those based on cloning and Sanger sequencing (Jaeger *et al.* 2014). For two of the three individuals, each of the alleles identified via 454 sequencing were also present in the cloning data (Table S5). However, several other alleles identified via cloning were absent from the 454-based genotypes. For the third individual, one allele was identified by both methods, but multiple alleles were present in only the cloning- or 454-based genotypes. Finally, based on qualitative comparison of MHC genotypes of three female Eastern Massasaugas and their respective offspring (*n* = 1, *n* = 7, *n* = 7), the transmission of MHC alleles was consistent with a Mendelian pattern of inheritance (Table S6). In addition to alleles present in the dams, each offspring possessed up to two putatively paternal alleles.

We applied strict filters to identify and remove artificial variants from the raw 454 sequencing data yielding accurate and unbiased MHC genotypes for a total of 154 Eastern Massasaugas. Nearly half of the raw reads generated by the five 454 sequencing runs of this study were discarded because they contained a mismatch in one of the primer sequences or did not show sufficient similarity to reference MHC sequences. Of the variants that passed this initial filtering step, a high proportion represented artefactual variants, presumably resulting from errors during amplification and sequencing. Although the use of proof-reading polymerases can minimize the rate of substitution errors, such enzymes can also increase the rate of recombination and can produce recombinant sequences when used with multi-allelic templates (Lenz & Becker 2008). Errors associated with 454 sequencing include point substitutions and (more frequently) insertion/deletion mutations (Margulies *et al.* 2005). Indeed, previous studies of MHC variation based on 454 sequencing have reported similar results to what we observed. For instance, ca. 70% of the variants identified by Galan et al. (2010) were observed just once in the dataset and were subsequently discarded, and ~80% of the variants identified by Huchard et al. (2012) were observed in only a single individual.

Lenz et al. (2008) described a protocol designed to minimize the formation of PCR artefacts; including (i) multiple independent reactions per sample, (ii) fewer PCR cycles (<25 cycles), (iii) longer extension time per cycle (>60 s), (iv) and a 'reconditioning' step in which PCR products from the first set of reactions are pooled, diluted, and used as template for a second round of PCR consisting of only a few cycles. Although we increased the extension time of each cycle, including multiple independent reactions for all samples was not feasible. In addition, the forward and reverse primers we used were degenerate and consisted of mixtures of 432 and 16 sequences, respectively (Kwok *et al.* 1994). Further, the low amount and quality of available Eastern Massasauga samples posed substantial challenges for amplification (Taberlet *et al.* 1999). Consequently, the two-step procedure we used was necessary to generate the amplicons for 454 sequencing, but did not permit us to implement every potential safeguard. Nevertheless, our analysis indicates no evidence of bias. Positive correlations between error rate and sequencing coverage have been reported (Huchard *et al.* 2012), with relatively more artefactual variants occurring in samples with more reads. In this study, the number of unique variants was independent from the sequencing coverage, indicating that my results were not biased in any systematic way (Sepil *et al.* 2012). As more genomic resources become available for snakes (Castoe *et al.* 2013; Vonk *et al.* 2013), future studies should seek to design less degenerate primers to improve amplification and minimize the formation of sequence artefacts.

The coverage (i.e., number of reads) yielded in a 454 sequencing run depends on several factors; including the concentration, purity, and length of the product, the size of picotiter plate, and the number of samples pooled within a single run (Brockman *et al.* 2008; Babik 2010). Here, the five sequencing runs generated hundreds to thousands of usable MHC reads *per sample*, permitting efficient population-level MHC genotyping (Fig. S2). Previous studies of MHC variation using similar methods have yielded varying coverage depths, from dozens (Babik *et al.* 2009; Erlich *et al.* 2011; Stiebens *et al.* 2013) to hundreds (Zagalska-Neubauer *et al.* 2010; Sepil *et al.* 2012; Promerova *et al.* 2012; Pavey *et al.* 2013; Lamaze *et al.* 2014) and even thousands (Huchard *et al.* 2012; Oomen *et al.* 2012) of reads per sample.

To filter artefactual variants and identify putatively true alleles, we retained only the variants with maximum-per amplicon frequencies (MPAF) greater than or equal to 10% within the pooled dataset (Fig. S1). Previous studies have employed similar methods, with MPAF thresholds ranging from 3–6% (Babik *et al.* 2009; Galan *et al.* 2010; Zagalska-Neubauer *et al.* 2010; Huchard *et al.* 2012; Oomen *et al.* 2012; Pavey *et al.* 2013). Our more conservative approach may have misidentified some true alleles as artefacts. However, simulations suggest that it is unlikely for true alleles to fall below this 10% threshold in any single individual (Galan *et al.* 2010). The frequency of each true allele is expected to be lower within individuals possessing more true alleles. Conversely, the frequency of each true allele should be somewhat higher within individuals possessing few true alleles. Therefore, rather than specify a single fixed threshold for all individuals, the threshold varied according to the frequency of the most common allele (Stiebens *et al.* 2013).

We compared MHC genotypes based on 454 sequencing with those derived from cloning/Sanger sequencing for three individuals (Jaeger *et al.* 2014). In two individuals, each of the alleles included in the 454-based genotype was also included in the cloning-based genotype. In the third individual, only one of three MHC-based alleles was also included in the cloning-based genotype. In each case, the cloning-based genotypes included more alleles than the 454-based genotypes. In contrast, Huchard et al. (2012) found 100% agreement between cloning- and 454-based genotypes for 5 individual Grey Mouse Lemurs (*Microcebus murinus*). In this case, variation was evaluated at individual MHC loci, which likely simplified interpretation of genotypes and contributed to the high level of agreement. To be sure, the 'two-PCR criterion' for identifying true alleles via cloning is not error-free (Babik *et al.* 2005; Kloch *et al.* 2010). This approach often underestimates true variation and may, in some cases, lead to the inclusion of artefactual alleles due to the unique errors associated with cloning (Lenz & Becker 2008; Promerova *et al.* 2012; Huchard *et al.* 2012). Finally, qualitative examination of MHC genotypes of three sets of dam-offspring dyads indicated a pattern consistent with Mendelian inheritance, with each offspring possessing between zero and two putatively paternal alleles. In a similar study, more than 200 mother-offspring dyads revealed >98% genotyping accuracy in the Grey Mouse Lemur (Microcebus murinus; Huchard *et al.* 2012). In each of the three litters we examined, at least one offspring genotype included fewer MHC alleles than the mother. While this may suggest imperfect genotyping methods, it may also be evidence of copy number variation or sharing of alleles across MHC loci (Lighten *et al.* 2014b).

The three approaches we used to evaluate the accuracy of the MHC genotyping method produced mixed results. More than half of the replicated samples yielded identical genotypes, with another third producing nearly identical genotypes. However, two of the 20 replicated genotypes differed markedly, possibly indicating cross-contamination between samples or book-keeping errors. While these results are comparable to the those of a previous study of MHC variation in the Great Tit (*Parus major*; Sepil *et al.* 2012), other studies have reported greater reproducibility in several rodent species (Galan *et al.* 2010), the Scarlet Rosefinch (*Carpodacus erythrinus*; Promerova *et al.* 2012), and the Blue Petrel (*Halobaena caerulea*; Strandh *et al.* 2012).

Ultimately, many factors can contribute to imperfect genotyping. Despite strict quality control procedures, samples may be cross-contaminated during amplification, library preparation, or 454 sequencing. Stochastic problems associated with low template quality and quantity may lead to allelic drop-out or drop-in, referring to the exclusion of true alleles and inclusion of artefactual alleles, respectively (Taberlet *et al.* 1996; Lighten *et al.* 2014a). While the Eastern Massasauga MHC genotypes obtained here may be conservative estimates of true variation, we found no evidence of any systematic biases that would potentially skew comparisons between populations.

**Literature Cited**

Babik, W. 2010. Methods for MHC genotyping in non-model vertebrates. Molecular Ecology Resources 10:237–251.

Babik, W., P. Taberlet, M. J. Ejsmond, and J. Radwan. 2009. New generation sequencers as a tool for genotyping of highly polymorphic multilocus MHC system. Molecular Ecology Resources 9:713–719.

Babik, W., W. Durka, and J. Radwan. 2005. Sequence diversity of the MHC DRB gene in the Eurasian beaver (*Castor fiber*). Molecular Ecology 14:4249–4257.

Brockman, W., P. Alvarez, S. Young, M. Garber, G. Giannoukos, W. L. Lee, C. Russ, E. S. Lander, C. Nusbaum, and D. B. Jaffe. 2008. Quality scores and SNP detection in sequencing-by-synthesis systems. Genome Research 18:763–770.

Castoe, T. A., A. P. J. de Koning, K. T. Hall, D. C. Card, D. R. Schield, M. K. Fujita, R. P. Ruggiero, J. F. Degner, J. M. Daza, W. Gu, J. Reyes-Velasco, K. J. Shaney, J. M. Castoe, S. E. Fox, A. W. Poole, D. Polanco, J. Dobry, M. W. Vandewege, Q. Li, R. K. Schott, A. Kapusta, P. Minx, C. Feschotte, P. Uetz, D. A. Ray, F. G. Hoffmann, R. Bogden, E. N. Smith, B. S. W. Chang, F. J. Vonk, N. R. Casewell, C. V. Henkel, M. K. Richardson, S. P. Mackessy, A. M. Bronikowski, A. M. Bronikowsi, M. Yandell, W. C. Warren, S. M. Secor, and D. D. Pollock. 2013. The Burmese python genome reveals the molecular basis for extreme adaptation in snakes. Proceedings of the National Academy of Sciences of the United States of America 110:20645–20650.

Erlich, R., X. Jia, S. Anderson, E. Banks, X. Gao, M. Carrington, N. Gupta, M. DePristo, M. Henn, and N. Lennon. 2011. Next-generation sequencing for HLA typing of class I loci. BMC Genomics 12:42.

Galan, M., E. Guivier, G. Caraux, N. Charbonnel, and J.-F. Cosson. 2010. A 454 multiplex sequencing method for rapid and reliable genotyping of highly polymorphic genes in large-scale studies. BMC Genomics 11:296.

Huchard, E., C. Albrecht, S. Schliehe-Diecks, A. Baniel, C. Roos, P. M. K. Peter, and M. Brameier. 2012. Large-scale MHC class II genotyping of a wild lemur population by next generation sequencing. Immunogenetics 64:895–913.

Jaeger, C. P., R. B. King, and M. R. Duvall. 2014. Initial characterization of major histocompatibility complex (MHC) class IIb exon 2 in an endangered rattlesnake, the eastern massasauga (*Sistrurus catenatus*). The Journal of North American Herpetology 2014:98–104.

Kloch, A., W. Babik, A. Bajer, E. Sinski, and J. Radwan. 2010. Effects of an MHC-DRB genotype and allele number on the load of gut parasites in the bank vole *Myodes glareolus*. Molecular Ecology 19:255–265.

Kwok, S., S. Y. Chang, J. J. Snisky, and A. Wang. 1994. A guide to the design and use of mismatched and degenerate primers. Genome Research 3:S39–S47.

Lamaze, F. C., S. A. Pavey, E. Normandeau, G. Roy, D. Garant, and L. Bernatchez. 2014. Neutral and selective processes shape MHC gene diversity and expression in stocked brook charr populations (*Salvelinus fontinalis*). Molecular Ecology 23:1730–1748.

Lenz, T. L., and S. Becker. 2008. Simple approach to reduce PCR artefact formation leads to reliable genotyping of *MHC* and other highly polymorphic loci – Implications for evolutionary analysis. Gene 427:117–123.

Lighten, J., C. van Oosterhout, and P. Bentzen. 2014a. Critical review of NGS analyses for de novo genotyping multigene families. Molecular Ecology 23:3957–3972.

Lighten, J., C. van Oosterhout, I. G. Paterson, M. McMullan, and P. Bentzen. 2014b. Ultra-deep Illumina sequencing accurately identifies MHC class IIb alleles and provides evidence for copy number variation in the guppy (*Poecilia reticulata*). Molecular Ecology Resources 14:753–767.

Margulies, M., M. Egholm, W. E. Altman, S. Attiya, J. S. Bader, L. A. Bemben, J. Berka, M. S. Braverman, Y.-J. Chen, Z. Chen, S. B. Dewell, L. Du, J. M. Fierro, X. V. Gomes, B. C. Godwin, W. He, S. Helgesen, C. H. Ho, C. H. Ho, G. P. Irzyk, S. C. Jando, M. L. I. Alenquer, T. P. Jarvie, K. B. Jirage, J.-B. Kim, J. R. Knight, J. R. Lanza, J. H. Leamon, S. M. Lefkowitz, M. Lei, J. Li, K. L. Lohman, H. Lu, V. B. Makhijani, K. E. McDade, M. P. McKenna, E. W. Myers, E. Nickerson, J. R. Nobile, R. Plant, B. P. Puc, M. T. Ronan, G. T. Roth, G. J. Sarkis, J. F. Simons, J. W. Simpson, M. Srinivasan, K. R. Tartaro, A. Tomasz, K. A. Vogt, G. A. Volkmer, S. H. Wang, Y. Wang, M. P. Weiner, P. Yu, R. F. Begley, and J. M. Rothberg. 2005. Genome sequencing in microfabricated high-density picolitre reactors. Nature 437:376–380.

Oomen, R. A., R. M. Gillett, and C. J. Kyle. 2012. Comparison of 454 pyrosequencing methods for characterizing the major histocompatibility complex of nonmodel species and the advantages of ultra deep coverage. Molecular Ecology Resources 13:103–116.

Pavey, S. A., M. Sevellec, W. Adam, E. Normandeau, F. C. Lamaze, P.-A. Gagnaire, M. Filteau, F. O. Hebert, H. Maaroufi, and L. Bernatchez. 2013. Nonparallelism in MHCIIβ diversity accompanies nonparallelism in pathogen infection of lake whitefish (*Coregonus clupeaformis*) species pairs as revealed by next-generation sequencing. Molecular Ecology 22:3833–3849.

Promerova, M., W. Babik, J. Bryja, T. Albrecht, M. Stuglik, and J. Radwan. 2012. Evaluation of two approaches to genotyping major histocompatibility complex class I in a passerine-CE-SSCP and 454 pyrosequencing. Molecular Ecology Resources 12:285–292.

Sepil, I., H. K. Moghadam, E. Huchard, and B. C. Sheldon. 2012. Characterization and 454 pyrosequencing of major histocompatibility complex class I genes in the great tit reveal complexity in a passerine system. BMC Evolutionary Biology 12:68.

Stiebens, V. A., S. E. Merino, F. J. Chain, and C. Eizaguirre. 2013. Evolution of MHC class I genes in the endangered loggerhead sea turtle (*Caretta caretta*) revealed by 454 amplicon sequencing. BMC Evolutionary Biology 13:95.

Strandh, M., H. Westerdahl, M. Pontarp, B. Canback, M. P. Dubois, C. Miquel, P. Taberlet, and F. Bonadonna. 2012. Major histocompatibility complex class II compatibility, but not class I, predicts mate choice in a bird with highly developed olfaction. Proceedings of the Royal Society of London. B Biological Sciences 279:4457–4463.

Taberlet, P., L. Waits, and G. Luikart. 1999. Noninvasive genetic sampling: look before you leap. Trends In Ecology & Evolution 14:323–327.

Taberlet, P., S. Griffin, B. Goossens, S. Questiau, V. Manceau, N. Escaravage, L. P. Waits, and J. Bouvet. 1996. Reliable genotyping of samples with very low DNA quantities using PCR. Nucleic Acids Research 24:3189–3194.

Vonk, F. J., N. R. Casewell, C. V. Henkel, A. M. Heimberg, H. J. Jansen, R. J. R. McCleary, H. M. E. Kerkkamp, R. A. Vos, I. Guerreiro, J. J. Calvete, W. Wüster, A. E. Woods, J. M. Logan, R. A. Harrison, T. A. Castoe, A. P. J. de Koning, D. D. Pollock, M. Yandell, D. Calderon, C. Renjifo, R. B. Currier, D. Salgado, D. Pla, L. Sanz, A. S. Hyder, J. M. C. Ribeiro, J. W. Arntzen, G. E. E. J. M. van den Thillart, M. Boetzer, W. Pirovano, R. P. Dirks, H. P. Spaink, D. Duboule, E. McGlinn, R. M. Kini, and M. K. Richardson. 2013. The king cobra genome reveals dynamic gene evolution and adaptation in the snake venom system. Proceedings of the National Academy of Sciences of the United States of America 110:20651–20656.

Zagalska-Neubauer, M., W. Babik, M. Stuglik, L. Gustafsson, M. Cichoń, and J. Radwan. 2010. 454 sequencing reveals extreme complexity of the class II major histocompatibility complex in the collared flycatcher. BMC Evolutionary Biology 10:395.

**Table S4.** Summary of replicated MHC IIB exon 2 genotypes (*n* = 20) determined via 454 sequencing in the Eastern Massasauga.

| **Individual** | **Result** | **Details** |
| --- | --- | --- |
| AZA.22898 | Identical | 7 alleles shared across replicates |
| AZA.41151 | Identical | 5 alleles shared across replicates |
| AZA.43327 | Identical | 5 alleles shared across replicates |
| AZA.45786 | Identical | 4 alleles shared across replicates |
| Cook.WS.12 | Identical | 4 alleles shared across replicates |
| Cook.22378 | Identical | 3 alleles shared across replicates |
| Cook.22380 | Identical | 3 alleles shared across replicates |
| Cook.Sica.JAR | Identical | 3 alleles shared across replicates |
| Clinton.Sica.832 | Identical | 3 alleles shared across replicates |
| AZA.41150 | Identical | 3 alleles shared across replicates |
| Cook.WS.07 | Identical | 2 alleles shared across replicates |
|  |  |  |
| Clinton.Sica.826 | Nearly identical | 2 alleles shared across replicates; 1 low-frequency allele in one replicate was present below threshold in other replicate |
| Cook.22299 | Nearly identical | 2 alleles shared across replicates; 1 low-frequency allele in one replicate was present below threshold in other replicate |
| Cook.22379 | Nearly identical | 3 alleles shared across replicates; 1 low-frequency allele in one replicate was present below threshold in other replicate |
|  |  |  |
| Clinton.Sica.825 | Similar | 4 alleles shared across replicates; 2 low-frequency alleles were not shared |
| Cook.Sica.11 | Similar | 3 alleles shared across replicates; 2 low-frequency alleles were not shared |
| Cook.Sica.1 | Similar | 3 alleles shared across replicates; 3 low-frequency alleles were not shared |
| Clinton.Sica.828 | Similar | 1 allele shared across replicates; 3 low-frequency alleles were not shared |
|  |  |  |
| Clinton.Sica.833 | Different | 3 alleles shared across replicates; 5 alleles were not shared (including some high-frequency alleles) |
| Clinton.Sica.827 | Different | 2 alleles shared across replicates; 6 alleles were not shared (including some high-frequency alleles) |

**Table S5.** Comparison of Eastern Massasauga MHC IIB exon 2 genotypes in three individuals based on cloning and Sanger sequencing and 454 sequencing. Alleles identified by both methods for each individual are highlighted.

| **Individual** | **Cloning & Sanger** | **454 Sequencing** |
| --- | --- | --- |
| Piatt.Sica.004 | *Sica*-DAB*03 | *Sica*-DAB*03 |
|  | *Sica*-DAB*04 | *Sica*-DAB*02 |
|  | *Sica*-DAB*06 | *Sica*-DAB*20 |
|  | *Sica*-DAB*07 |  |
|  | *Sica*-DAB*08 |  |
|  | *Sica*-DAB*01 |  |
|  |  |  |
| Piatt.Sica.033 | *Sica*-DAB*01 | *Sica*-DAB*01 |
|  | *Sica*-DAB*02 | *Sica*-DAB*02 |
|  | *Sica*-DAB*03 | *Sica*-DAB*03 |
|  | *Sica*-DAB*04 |  |
|  | *Sica*-DAB*05 |  |
|  | *Sica*-DAB*06 |  |
|  |  |  |
| Piatt.Sica.034 | *Sica*-DAB*01 | *Sica*-DAB*01 |
|  | *Sica*-DAB*02 | *Sica*-DAB*02 |
|  | *Sica*-DAB*03 | *Sica*-DAB*03 |
|  | *Sica*-DAB*04 | *Sica*-DAB*04 |
|  | *Sica*-DAB*05 |  |
|  | *Sica*-DAB*06 |  |
|  | *Sica*-DAB*08 |  |

**Table S6.** MHC IIB exon 2 genotypes of three Eastern Massasauga mothers and their offspring. The prefix *'Sica*-DAB*' is omitted from allele names for brevity. The presence of a given maternal allele in an offspring genotype is indicated with a plus sign (+). Putative paternal alleles (alleles not found in mothers) are also listed for each offspring.

| **Individual** |  | **Maternal Alleles** | | | | | **Paternal Alleles** |
| --- | --- | --- | --- | --- | --- | --- | --- |
| Piatt.Sica.002 | Dam | *02 | *03 | *21 | *23 | *25 |  |
| Piatt.Sica.003 | Offspring |  | + |  |  | + |  |
|  |  |  |  |  |  |  |  |
| Piatt.Sica.004 | Dam | *02 | *03 | *20 |  |  |  |
| Piatt.Sica.006 | Offspring |  | + | + |  |  | *12 |
| Piatt.Sica.007 | Offspring | + | + | + |  |  |  |
| Piatt.Sica.008 | Offspring | + | + | + |  |  | *22 |
| Piatt.Sica.009 | Offspring | + | + | + |  |  |  |
| Piatt.Sica.010 | Offspring |  | + | + |  |  | *24 |
| Piatt.Sica.011 | Offspring | + | + | + |  |  | *22 |
| Piatt.Sica.012 | Offspring |  | + | + |  |  | *24 |
|  |  |  |  |  |  |  |  |
| Piatt.Sica.016 | Dam | *02 | *03 | *20 | *24 |  |  |
| Piatt.Sica.017 | Offspring |  | + | + |  |  |  |
| Piatt.Sica.019 | Offspring |  | + | + | + |  | *28 |
| Piatt.Sica.020 | Offspring |  | + | + | + |  | *18 |
| Piatt.Sica.022 | Offspring |  | + | + | + |  | *19, *23 |
| Piatt.Sica.023 | Offspring |  | + | + | + |  | *28 |
| Piatt.Sica.024 | Offspring | + | + |  |  |  | *19, *23 |
| Piatt.Sica.026 | Offspring |  | + | + | + |  |  |

**Figures**

**Figure S1.** Eastern Massasauga MHC IIB exon 2 variants with maximum per-amplicon frequencies above 0.10 (10%; red dotted line) were considered putatively true alleles, while those below this threshold were treated as artefacts and excluded from subsequent analyses. This resulted in 25 putatively true alleles, representing ~75% of the total reads. Note that overlapping points are indicated with darker shading.

**Figure S2.** The number of MHC IIB exon 2 alleles was not significantly correlated with sequencing depth (number of reads) per individual (*n* = 169, *r* = –0.063, *P* = 0.416). Note that overlapping points are indicated with darker shading.
